# Bilateral pneumothoraces complicating reduction mammoplasty: a case report

**DOI:** 10.1186/1471-2482-13-29

**Published:** 2013-07-26

**Authors:** Stylianos Mavridis, Hans Georg Gnauk, Martina Schumacher, Roland Wagner

**Affiliations:** 1 Vascular and Thoracic Surgery Department, Ernst von Bergmann Clinic, Charlottenstraße 72, Potsdam, 14467, Germany

**Keywords:** Pneumothorax, Bilateral, Mammoplasty

## Abstract

**Background:**

Bilateral pneumothoraces after cosmetic breast surgery are rare and sporadically reported in the literature.

**Case presentation:**

A 65-year-old female patient developed bilateral pneumothoraces after bilateral breast reduction surgery. Emergent chest tube thoracostomy was performed on both sides. The chest drains were removed on the fourth day (left side) and sixth day (right side), and the patient was discharged after 7 days of hospitalization without any further complications.

**Conclusion:**

To our knowledge, the English-language literature contains no other reports of bilateral pneumothoraces after reduction mammoplasty.

## Background

Vincenz Czerny was the first to perform cosmetic breast surgery in 1895. Since then, aesthetic mammoplasty has become a widely performed procedure in plastic surgery.

Hematoma, seroma, extrusion, and pneumothorax are typical early complications of cosmetic breast surgery, whereas only a few cases of bilateral pneumothoraces, isolated subcutaneous emphysema, or pericardial effusion have been reported [1-4]. In 2005, Osborn and Stevenson [3] demonstrated that the occurrence of pneumothorax after mammoplasty is underestimated. In most cases, the etiology is iatrogenic due to local anesthetic needle injection or intraoperative laceration of the intercostal fascia or pleura. Other potential related causes are barotrauma after high-pressure ventilation or intubation maneuvers, increased air pressure in the surgical cavity secondary to the advancing implant, or pre-existing lung blebs and bullae [2,3,5].

We searched the English-language literature and located previous reports of bilateral pneumothoraces after augmentation mammoplasty, but found no cases involving bilateral pneumothoraces complicating breast reduction surgery. Consequently, we assume that the first such case is herein described.

## Case presentation

A 65-year-old female patient was scheduled for an elective bilateral breast reduction surgery due to chronic back pain and hypertrophic and ptotic breasts in a private clinic for aesthetic surgery in Potsdam. She had a past medical history of smoking; hypertension; hypercholesterolemia; cervical disc protrusions at C2/C3, C5/C6, and C6/C7; intervertebral thoracic disc herniation at T7/T8 and protrusions from T8 to T12; mild lumbar protrusions at L3/L4 and L4/L5; and disc herniation at L5/S1. These disc abnormalities had restrained the patient’s movement for the past 2 years, and she was confined to a wheelchair. She also suffered from left-sided de Quervain’s tendinitis and gastric ulceration.

Surgery was performed under general anesthesia. A total of 150 ml of a local anesthetic combined with adrenaline was injected on each side, and the bilateral procedure was continued using the inverted T-pattern. The total amount of reduced breast tissue, including liposuction, comprised 320 g on the right side (47% fat) and 392 g on the left side (38.3% fat). At the end of surgery, a 12-ch Redon drain was placed on each side. She was uneventfully extubated and moved into the recovery room.

**Figure 1:**
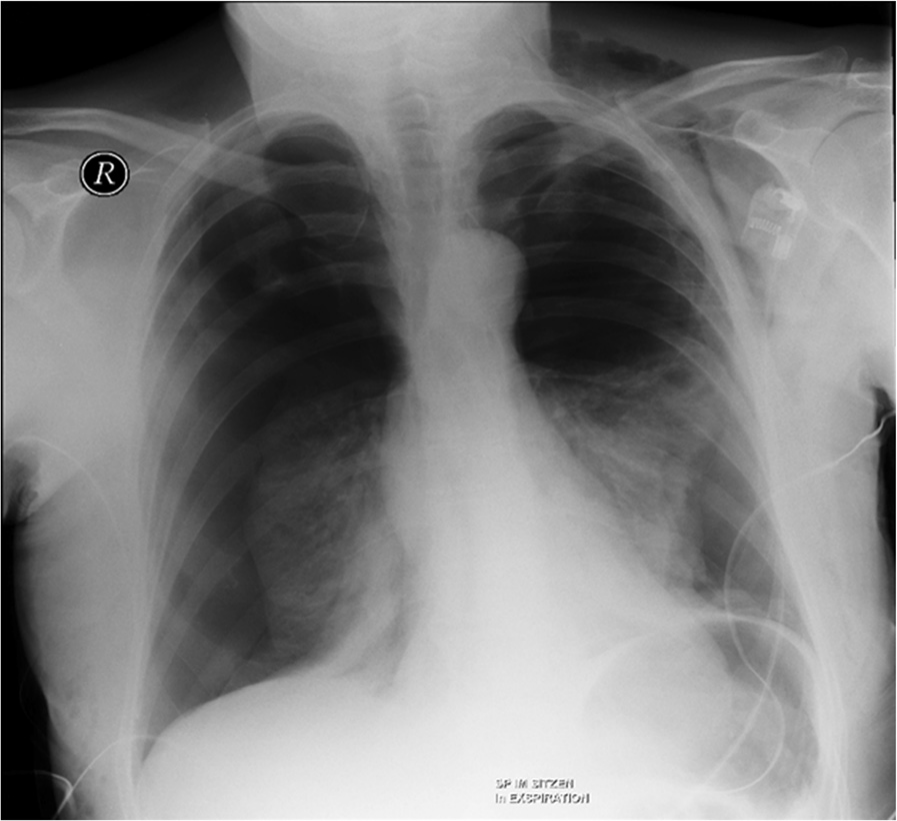
Chest radiograph at time of admission demonstrating the bilateral pneumothoraces after reduction mammoplasty.

Postoperatively, she experienced acute shortness of breath at rest with a drop in oxygen saturation. Pneumothorax was suspected, and she was immediately transferred by paramedic service to the emergency room of our institution. Upon admission, she demonstrated severe dyspnea, and clinical examination revealed cyanosis, abnormal ventilation of both sides with normal and rapid heart sounds, and bilateral hyperresonant percussion. Her blood pressure was 100/70 mmHg, heart rate was rhythmic at 120/min, body temperature was normal at 36.6°C, and oxygen saturation was 65% on room air. A chest radiograph revealed bilateral pneumothoraces with collapsed lungs but without mediastinal shift or diaphragm depression (Figure 1).

**Figure 2:**
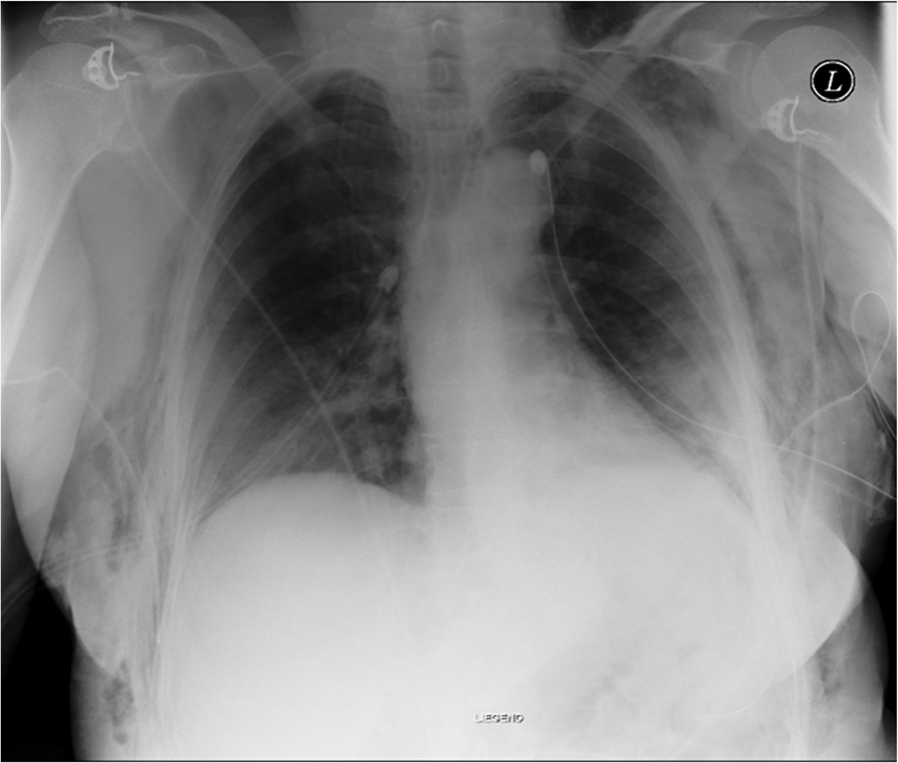
Post-tubing state.

Emergent chest tube thoracostomy with 24-ch thoracic drains was performed on both sides, starting on the right. After local anesthetic injection, the chest tubes were inserted bilaterally in the seventh intercostal space at the mid-axillary line and attached to an underwater seal. A subsequent chest radiograph confirmed re-expansion of both lungs, although apical pneumothorax of 2 cm was still noticeable at rest on the right side (Figure 2). On the third day, moderate right-sided subcutaneous emphysema was observed, and the right drain was consequently placed on suction.

The chest drains were removed on the fourth day (left side) and sixth day (right side) after 2 days without air leakage and evaluation of a control chest radiograph with a clamped drain. The subsequent clinical course was uneventful, and the patient was discharged after 7 days of hospitalization without any further complications.

## Conclusions

In the field of plastic surgery, pneumothorax complicating breast aesthetic surgery (and more specifically, augmentation mammoplasty) was historically thought to be rare.

In 2005, a survey of the California Society of Plastic Surgeons conducted by Osborn and Stevenson [3] showed that one-third of participating plastic surgeons had experienced at least one case of pneumothorax after breast augmentation. The precise etiology was not easy to determine. In most cases, the pneumothorax was iatrogenically caused by the surgeon (e.g., lung penetration during local anesthetic injection, unintentional laceration of the pleura, or injury to the intercostal fascia) or the anesthetist (e.g., high-pressure ventilation or intubation maneuvers).

Also in 2005, Fayman et al. [5] proposed another mechanism that may induce pneumothorax after augmentation mammoplasty: elevated air pressure in the surgical pocket generated from the advancing breast implant, resulting in the entrance of air into the pleural cavity. Pre-existing pulmonary pathology such as bullae, blebs, or emphysema can also be responsible.

On the other hand, pneumothorax occurs almost daily in the field of thoracic surgery, and thoracostomy is the most frequently performed thoracic procedure. Bilateral pneumothoraces are rarer. They can be traumatic, iatrogenic, secondary to underlying interstitial pulmonary disease (e.g., pneumonia, emphysema, pulmonary metastases, etc.), or even spontaneous. Bilateral tension pneumothoraces occur when the degree of collapse of both lungs is similar on a chest radiograph without tracheal shift because of the bilateral pathology [6]. A bilateral “deep sulcus sign” with depression of both diaphragms can also been detected [7].

Bilateral tension pneumothorax represents an emergency situation. Affected patients present with severe dyspnea, cyanosis, or tachycardia that leads to decreased cardiac output because venous return is compromised due to compression of the vena cava, right atrium, or large veins, which can be potentially life-threatening [6,8]. Therefore, prompt treatment is required. Needle decompression can be performed followed by percutaneous catheter insertion or thoracostomy.

In our case, emergent bilateral thoracostomy was performed using two 24-ch chest drains, one on each side. The clinical state of the patient allowed us to perform thoracostomy in the emergency room rather than needle decompression. Other colleagues confronted with intraoperative tension pneumothorax and the need for intraoperative resuscitation first carried out needle decompression and secondarily inserted chest drains. In such cases, the lack of time favors emergent needle decompression. We preferred to insert a medium-diameter chest drain in the seventh intercostal space at the mid-axillary line rather than in the second intercostal space at the mid-clavicular line to achieve a better postoperative aesthetic result. We do not believe that a chest tube of a smaller or larger diameter or another tube insertion location would have changed the outcome or would have had an effect on the total duration of the hospital stay. The chest drains were not directly placed on suction to avoid pulmonary expansion edema. No persistent air leakage from the thoracic drains was observed, so no further radiological examination or surgical procedure was necessary. The pneumothoraces resolved uneventfully over the next several days.

We assume that the cause of the pneumothoraces in the present case was iatrogenic. However, considering the patient’s age and smoking history, there is a chance that spontaneous bilateral pneumothoraces resulted from blebs or bullae that ruptured during the operation.

In summary, we suggest that patients should always be informed of the risk of pneumothorax before aesthetic breast surgery and that surgeons should be aware of its symptoms and be prepared for emergency treatment. Thoracic surgical procedures always have the potential to be complicated by pneumothorax.

## Consent

Written informed consent was obtained from the patient for publication of this Case report and any accompanying images. A copy of the written consent is available for review by the Editor of this journal.

## Competing interests

The authors declare that they have no competing interests.

## Authors’ contributions

SM designed the case report, collected the data, and wrote sections of the manuscript. HGG analyzed and interpreted the data. MS designed the study and wrote sections of the manuscript. RW supervised the study and led scientific discussion. All authors read and approved the final manuscript.

## Pre-publication history

The pre-publication history for this paper can be accessed here:

http://www.biomedcentral.com/1471-2482/13/29/prepub
